# Decoding cardiovascular risks: analyzing type 2 diabetes mellitus and ASCVD gene expression

**DOI:** 10.3389/fendo.2024.1383772

**Published:** 2024-04-23

**Authors:** Youqi Zhang, Liu Ji, Daiwei Yang, Jianjun Wu, Fan Yang

**Affiliations:** ^1^ Department of Cardiology, The Second Affiliated Hospital of Harbin Medical University, Harbin, China; ^2^ Key Laboratory of Myocardial Ischemia, Ministry of Education, Harbin, China; ^3^ Department of Orthopedics, The Fourth Affiliated Hospital of Harbin Medical University, Harbin, China; ^4^ State Key Laboratory of Component-based Chinese Medicine, Tianjin University of Traditional Chinese Medicine, Tianjin, China

**Keywords:** type 2 diabetes mellitus, atherosclerosis, NHANES database, GEO database, WGCNA

## Abstract

**Background:**

ASCVD is the primary cause of mortality in individuals with T2DM. A potential link between ASCVD and T2DM has been suggested, prompting further investigation.

**Methods:**

We utilized linear and multivariate logistic regression, Wilcoxon test, and Spearman’s correlation toanalyzethe interrelation between ASCVD and T2DM in NHANES data from 2001-2018.The Gene Expression Omnibus (GEO) database and Weighted Gene Co-expression Network Analysis (WGCNA) wereconducted to identify co-expression networks between ASCVD and T2DM. Hub genes were identified using LASSO regression analysis and further validated in two additional cohorts. Bioinformatics methods were employed for gene ontology and Kyoto Encyclopedia of Genes and Genomes (KEGG) pathway enrichment analysis, along with the prediction of candidate small molecules.

**Results:**

Our analysis of the NHANES dataset indicated a significant impact of blood glucose on lipid levels within diabetic cohort, suggesting that abnormal lipid metabolism is a critical factor in ASCVD development. Cross-phenotyping analysis revealed two pivotal genes, ABCC5 and WDR7, associated with both T2DM and ASCVD. Enrichment analyses demonstrated the intertwining of lipid metabolism in both conditions, encompassing adipocytokine signaling pathway, fatty acid degradation and metabolism, and the regulation of adipocyte lipolysis. Immune infiltration analysis underscored the involvement of immune processes in both diseases. Notably, RITA, ON-01910, doxercalciferol, and topiramate emerged as potential therapeutic agents for both T2DM and ASCVD, indicating their possible clinical significance.

**Conclusion:**

Our findings pinpoint ABCC5 and WDR7 as new target genes between T2DM and ASCVD, with RITA, ON-01910, doxercalciferol, and topiramate highlighted as promising therapeutic agents.

## Introduction

Type 2 Diabetes Mellitus (T2DM) constitutes a profound global health dilemma, significantly amplifying the risk of morbidity and mortality related to atherosclerosis cardiovascular disease (ASCVD) (1). This ailment drastically diminishes life expectancy, evidenced by findings that, compared to individuals without diabetes, men and women afflicted by diabetes mellitus experience a reduction in lifespan of approximately 7.5 and 8.2 years, respectively. The anticipated growth of the global diabetic population to approximately 439 million adults by 2030 underscores a 69% increase in developing countries and a 20% increase in developed countries (2).Addressing this imminent crisis necessitates a large-scale, population-based follow-up study vital for prevention, early detection, and the identification of associated risk factors.

T2DM patients represent distinctive cardiovascular profiles marked by elevated atherosclerotic plaque burdens, larger atheromatous plaque volumes, and lipid metabolism dysfunction (3–5). Decades ago, the groundbreaking Framingham Heart Study highlighted the prospective link between diabetes mellitus and an increased prevalence of cardiovascular disease, particularly impactful in women across various age groups (6). Despite the historical recognition of heightened risks, significant progress in improving cardiovascular outcomes through glucose reduction has remained elusive. Hafner and colleagues (7) delved into the mortality landscape within cardiovascular diseases among T2DM patients, revealing a concerning outlook. The mortality rate for T2DM patients without a history of myocardial infarction (MI) is 15.4%. This rate increases dramatically to 42.0% for T2DM patients with a history of MI. In stark contrast, individuals without T2DM face significantly lower risks, with mortality rates from cardiovascular causes at 2.1% and 15.9% for those without and with a history of MI, respectively.

In recent years, the explosion of genomic data availability has elevated bioinformatics analysis methods to indispensable tools in scientific research (8, 9). Bioinformatics analysis plays a pivotal role in deciphering this wealth of information, enabling the identification of Differentially Expressed Genes (DEGs), conducting intricate Gene Ontology (GO) analyses, and performing insightful pathway analyses (10). In our study, we have seamlessly integrated cutting-edge bioinformatics techniques with data sourced from two pivotal databases: the National Health and Nutrition Examination Survey (NHANES) and the Gene Expression Omnibus (GEO) hosted by the National Center for Biotechnology Information (NCBI). The combination of these two databases allows the complex relationship between T2DM and ASCVD to be elucidated at the metabolic and molecular levels. We aim to employ a multifaceted bioinformatics approach to unravel the genetic mechanisms underpinning the comorbidity of T2DM and ASCVD.

## Materials and Methods

### Data Collection

Our study samples and data were sourced from the NHANES (https://wwwn.cdc.gov/nchs/nhanes/) from 2001 to 2018. NHANES is a nationally representative survey of the non-institutionalized civilian population in the US, and the survey involved interviews conducted at participants’ homes and standardized physical examinations, including laboratory tests, performed at mobile screening centers (MEC).

**Figure 1:**
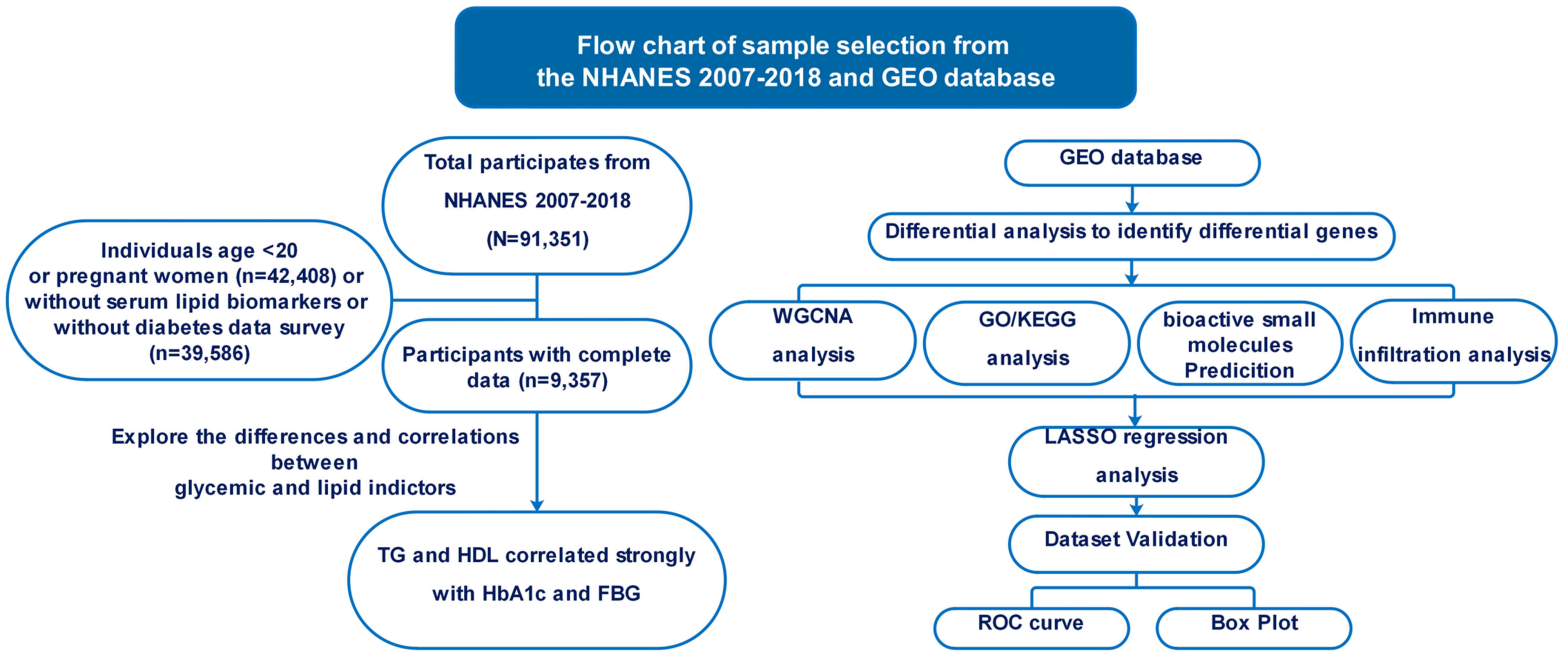
Flowchart illustrating the methodology employed in this study.

To identify pertinent datasets for our investigation, we conducted a comprehensive search of the NCBI GEO database (https://www.ncbi.nlm.nih.gov/geo/) using specific medical keywords such as “Type 2 diabetes mellitus”, “Atherosclerosis”, “Homo sapiens”, “Expression profiling by array”, and “expression profiling analysis”. The objective was to pinpoint datasets that met stringent criteria: they had to contain archived information on both case and control groups, offer raw data for further analysis, and enable expression analysis using array methods. Additionally, our search was limited to datasets exclusively featuring data from Homo sapiens (**Figure 1**).

Microarray analysis was performed using two different platforms: the GSE40231 dataset employed the GPL570 (Affymetrix Human Genome U133 Plus2 microarrays), whereas the GSE9006 dataset utilized the GPL96 (Illumina HumanHT-12 V4.0 Expression Bead Chip). The T2DM dataset (GSE9006) comprised gene expression data from peripheral blood mononuclear cells (PBMC) collected from 24 healthy people and 12 newly diagnosed with T2DM. We utilized 40 samples of Atherosclerotic Arterial Wall (AAW) and 40 samples of Non-Atherosclerotic Arterial Wall (NAW) from GSE40231 to identify DEGs, including Differentially Expressed mRNAs (DEmRNAs) and Long Non-Coding RNAs (DElncRNAs). Additionally, we validated the diagnostic efficacy of essential genes using datasets from two different platforms: the T2DM (GSE71416) from the GPL570 platform, which included 14 morbidly obese diabetic patients (cases) and six morbidly obese non-diabetic patients, and the AS dataset (GSE43292) utilizing 32 AAW samples and 32 NAW samples.

### Study population

In this cohort study, we selected adult participants from the NHANES spanning from 2001 to 2018, totaling 91,351 individuals. The inclusion criteria mandated participants to be at least 20 years old and not pregnant, narrowing down the cohort to 48,943 participants. Following the exclusion of individuals with missing data on fasting blood glucose (FBG), hemoglobin A1c (HbA1c), triglyceride (TG), total cholesterol, low-density lipoprotein (LDL), and high-density lipoprotein (HDL), the final participant was 9,357.

To identity individuals withT2DM, we adhered to the American Diabetes Association’s diagnostic criteria, which include:(1)a self-reported physician diagnosis of diabetes; (2) the use of oral hypoglycemic agents or insulin for treatment; (3) a fasting plasma glucose level of at least 126 mg/dL; (4) an HbA1c level of at least 6.5%. Following these criteria, 1,829 participants were classified into the diabetes group, whereas 7,528 were allocated to the control group. Our study rigorously followed the Strengthening the Reporting of Observational Studies in Epidemiology (STROBE) reporting guidelines to ensure the highest level of clarity, transparency, and rigor in reporting the observational study findings.

### Differential analysis

We used R software version 4.2.2 and processed raw matrices downloaded from the datasets. Data normalization was performed using the RMA algorithm after preprocessing and converting probe IDs to gene symbols using annotated platform files. Empty probes were removed, and values for genes with multiple probes were averaged to enhance result reliability.

Separate analyses were conducted for AS and T2DM datasets. The limma package was employed with stringent criteria (|logFC| >1 and Padj< 0.05) to identify DEGs. This approach identified genes with significant expression changes between case and control groups. An overlap analysis of DEGs from T2DM and AS datasets was also performed. We identified common DEGs to uncover potential molecular links between T2DM and AS. Venn Analytics was used for this analysis, allowing for a comprehensive evaluation of shared genes (11). These overlapping DEGs form the basis for further exploration into the molecular mechanisms connecting T2DM and AS.

### Functional Enrichment Analysis

We conducted an integrated analysis using Gene Ontology (GO) and Kyoto Encyclopedia of Genes and Genomes (KEGG) to explore biological functions and pathways associated with the identified genes (12–14). Visual representations were generated using the ggplot2 package in R4.2.2, facilitating a clear understanding of enriched pathways and their significance. Pathways with a P-value < 0.05 were considered statistically significant, indicating robust associations between T2DM and AS.

### Construction of WGCNA co-expression modules for datasets

We applied weighted gene co-expression network analysis (WGCNA) to assess gene expression patterns in extensive T2DM and AS datasets (15). Genes with significant Padj values (P <0.05) and absolute logarithmic changes greater than 1 were chosen. Combining the soft thresholding-derived neighbor-joining matrix with a gene-gene correlation matrix,we explored gene connectivity, describing the network’s interconnectedness. Co-expression modules were identified through transformation of the neighbor-joining matrix into a topological overlap matrix, followed by gene hierarchical clustering dendrogram analysis, grouping genes with similar expression patterns and implying potential functional links. To pinpoint clinically relevant modules, we calculated module eigengene sand examined their correlation with clinical features, focusing on modules with positive correlations in both T2DM and AS datasets. Positive correlations between modules and diseases indicated strong associations between module genes and the respective disease.

### Identification of critical genes

LASSO (Least Absolute Shrinkage and Selection Operator) is a regression-based methodology that accommodates many covariates in the model. Notably, LASSO possesses a distinct feature of penalizing the absolute value of regression coefficients. Our study employed the ‘glmnet’ package in the R software to conduct LASSO analysis on the candidate hub genes and DEGs. This analysis aided in identifying the final hub genes that exhibited strong associations with the studied conditions.

### Diagnostic potency assessment of Hub genes and their expression correlation

To assess the potential diagnostic utility of the hub genes, we evaluated performance using the T2DM dataset (GSE9006 and GSE71416) and the AS dataset (GSE40231 and GSE43292). The ROC curves provide a graphical representation of the sensitivity and specificity of the hub genes as diagnostic markers. By analyzing area under curve (AUC), we can measure the accuracy with which central genes classify disease and control groups. The closer the AUC value is to 1, the higher the diagnostic accuracy.

Additionally, to investigate significant differences in gene expression levels between the groups, we employed t-tests. These tests allowed us to compare the expression levels of the hub genes in individuals with T2DM, AS, and controls.

### Immune Cell Composition

The CIBERSORT algorithm was employed to calculate the proportions of various immune cells in the peripheral blood of patients with T2DM and non-T2DM participants and the arterial wall of patients with AS and non-AS participants. Using the R package “CIBERSORT” and the expression matrices, we determined the proportions of 22 immune cell types in the T2DM and AS disease groups and their respective control groups. To visually represent the proportions of the 22 immune cells in the disease and control groups for T2DM and AS, we generated heatmaps using the “corrplot” package. These heat maps provided a comprehensive view of the quantitative correlations between each disease condition’s different immune cell types. Additionally, we employed the “ggplot2” Rpackage to explore potential associations between immune cell proportions and the expression levels of specific diagnostic markers in the context of T2DM and AS.

### Target prediction of bioactive small molecules

The Connectivity Map (cMAP) database provided by the Broad Institute (https://clue.io)consist of drug-like compounds tested for gene expression (16). We uploaded all common DEGs in the GSE9006 and GSE40231 datasets to the cMAP database to screen small molecule candidates. We screened with a score greater than 90, suggesting they potentially have therapeutic effects on T2DM and AS.

### Statistical analysis

All statistical analyses were done using R software (R version 4.2.2). Means and confidence intervals for quartile HbA1c and quartile FBG versus lipid indices were calculated by linear regression. Wilcoxon test was used for statistical analysis between diabetic and non-diabetic groups. We used Spearman’s correlation analysis to investigate the relationship between glycemia and lipid indices by calculating the means and confidence intervals of quartile HbA1c and quartile FBG versus lipid indices in the T2DM using multivariate logistic regression modeling. Statistical significance was determined based on P-values less than 0.05, 0.01, or 0.001. These thresholds helped identify genes with statistically significant differences in expression levels between the disease and control groups. By combining ROC curve analysis and t-tests, we can assess the diagnostic performance of the pivotal genes and determine which genes may be promising biomarkers for differentiating between T2DM, AS, and controls. P-values less than 0.05 (P < 0.05) indicate statistical significance. Significance levels are expressed as follows: *P < 0.05, **P < 0.01, ***P < 0.001. ***P < 0.001.

## Results

### Participant Characteristics

**Table 1** shows the demographic characteristics of the 9,357 participants included in the study, segregated into two groups: 1,829 individuals diagnosed with T2DM and 7,528 controls. The prevalence of T2DM was significantly higher in males (P=0.026), and the group of former smokers, hypertensive, alcohol drinkers, and less educated had a higher risk of developing the disease (P < 0.001). Patients with T2DM had a lower BMI than the healthy population (P < 0.001).

**Table 1:** Baseline characteristics of study population, NHANES 2001–2018.

Characteristics	ALL (N=9,357)	Non-Diabetes (N=7,528)	Diabetes (N=1,829)	OR	P value
Age	50 [35,65]	46 [33,62]	63 [53,72]	1.05 [1.04,1.05]	<0.001
Sex					0.026
Female	4657 (49.8%)	3790 (50.3%)	867 (47.4%)	Ref.	
Male	4700 (50.2%)	3738 (49.7%)	962 (52.6%)	1.12 [1.02,1.25]	
Ethnicity					<0.001
Non-Hispanic black	1911 (20.4%)	1489 (19.8%)	422 (23.1%)	Ref.	
Mexican American	1665 (17.8%)	1288 (17.1%)	377 (20.6%)	1.03 [0.88,1.21]	
other	892 (9.53%)	701 (9.31%)	191 (10.4%)	0.96 [0.79,1.17]	
other Hispanic	612 (6.54%)	458 (6.08%)	154 (8.42%)	1.19 [0.96,1.47]	
Non-Hispanic white	4277 (45.7%)	3592 (47.7%)	685 (37.5%)	0.67 [0.59,0.77]	
Smoke					<0.001
never	4920 (52.6%)	4033 (53.6%)	887 (48.5%)	Ref.	
now	1963 (21.0%)	1652 (21.9%)	311 (17.0%)	0.86 [0.74,0.99]	
once	2474 (26.4%)	1843 (24.5%)	631 (34.5%)	1.56 [1.39,1.75]	
Bp					<0.001
no	6059 (64.8%)	5399 (71.7%)	660 (36.1%)	Ref.	
yes	3298 (35.2%)	2129 (28.3%)	1169 (63.9%)	4.49 [4.03,5.00]	
Alcohol					<0.001
heavy	4948 (52.9%)	4210 (55.9%)	738 (40.3%)	Ref.	
moderate	3253 (34.8%)	2438 (32.4%)	815 (44.6%)	1.91 [1.71,2.13]	
no	1156 (12.4%)	880 (11.7%)	276 (15.1%)	1.79 [1.53,2.09]	
Education					<0.001
College	4824 (51.6%)	4025 (53.5%)	799 (43.7%)	Ref.	
High school	2204 (23.6%)	1763 (23.4%)	441 (24.1%)	1.26 [1.11,1.43]	
Less than high school	2329 (24.9%)	1740 (23.1%)	589 (32.2%)	1.71 [1.51,1.92]	
Poverty income ratio					<0.001
<=1.0	1685 (18.0%)	1310 (17.4%)	375 (20.5%)	Ref.	
1.1-3.0	4027 (43.0%)	3171 (42.1%)	856 (46.8%)	0.94 [0.82,1.08]	
>3.0	3645 (39.0%)	3047 (40.5%)	598 (32.7%)	0.69 [0.59,0.79]	
Antilipemic drugs					<0.001
no	5762 (61.6%)	5268 (70.0%)	494 (27.0%)	Ref.	
yes	3595 (38.4%)	2260 (30.0%)	1335 (73.0%)	6.30 [5.62,7.07]	
Antidiabetic drugs					<0.001
no	8328 (89.0%)	7528 (100%)	800 (43.7%)	Ref.	
yes	1029 (11.0%)	0 (0.00%)	1029 (56.3%)		
BMI (kg/m2)	29.0 (6.9)	28.3 (6.5)	31.9 (7.6)	1.07 [1.06,1.08]	<0.001

Data are numeric (percentages), or median [interquartile spacing]. All estimates take into account the complex survey design. Bp, blood pressure; BMI, body mass index.

### Association between Baseline glycemic and lipid markers

**Figure 2:**
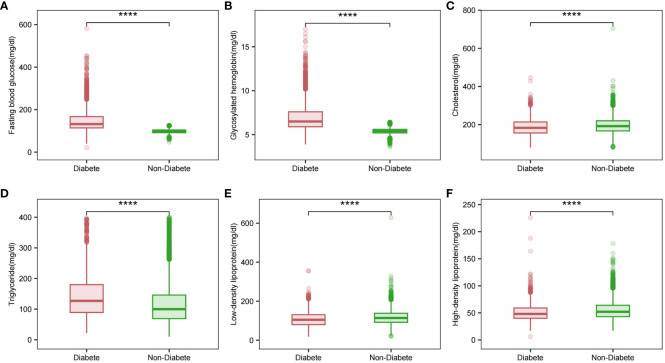
Glycemic and lipid profiles in different diabetic states. **(A)** Fasting glucose profile in different diabetic states; **(B)** Glycosylated hemoglobin profile in different diabetic states; **(C)** Total cholesterol profile in different diabetic states; **(D)** Triglyceride profile in different diabetic states; **(E)** LDL profile in different diabetic states; **(F)** HDL profile in different diabetic states. Comparison of means between groups performed by wilcox test (****P <0.0001).

**Figure 3:**
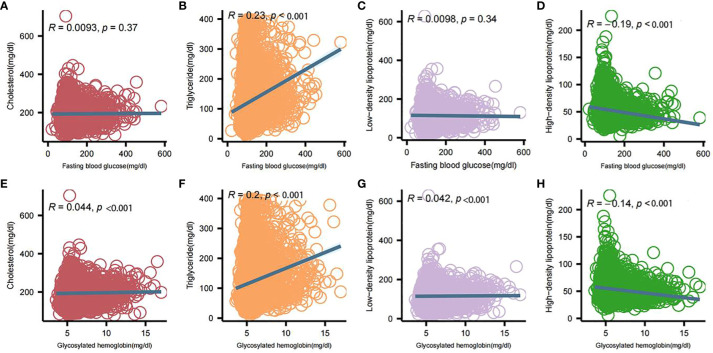
Correlation analysis between blood glucose and lipid profile components. **(A)** Correlation of fasting glucose and total cholesterol; **(B)** Correlation of fasting glucose and triglycerides; **(C)** Correlation of fasting glucose and low-density lipoproteins; **(D)** Correlation of fasting glucose and high-density lipoproteins; **(E)** Correlation of glycosylated hemoglobin and total cholesterol; **(F)** Correlation of glycosylated hemoglobin and triglycerides; **(G)** Correlation of glycosylated hemoglobin and low-density lipoproteins; **(H)** Correlation between glycosylated hemoglobin and high-density lipoprotein. Intergroup correlations were determined using spearman.

Significant differences in HDL, LDL, TG, total cholesterol, FPG, and HbA1c levels between the two groups of patients were analyzed by comprehensive generalized linear regression (**Figures 2A-F**). FBG, HbA1c, and TG levels were significantly higher in diabetic patients than in non-diabetic patients (P < 0.001) (**Figures 2A, B, D**). In addition, total cholesterol, LDL, and HDL levels were significantly lower in diabetic patients than non-diabetic patients(**Figures 2C, E, F**). In **Figure 3**, our analysis showed a significant positive correlation (P < 0.01) between FBG, HbA1c, and TG (**Figures 3B, F**). In contrast, there was a significant negative correlation (P < 0.01) between FBG, HbA1c, and HDL levels (**Figures 3D, H**). There was also a low positive correlation (P < 0.01) between HbA1c and total cholesterol, LDL (**Figures 3E, G**). Meanwhile, FBG also showed a lower positive correlation with total cholesterol and LDL as well (**Figures 3A, C**). These findings highlight the unique lipid profile of diabetic patients and emphasize the intricate relationship between glycemic control and lipid metabolism.

### Glycemic and Lipid Indices Correlation in a Diabetic Cohort

**Table 2** stratifies FBG into quartiles, revealing a progressive increase in TG with higher FBG levels, indicating a heightened risk (OR 1.4, 95% CI [1.3-1.5]). Concurrently, HDL levels inversely correlate with FBG (P < 0.001). Similarly, **Table 3** categorizes HbA1c into intervals, demonstrating significant TG elevation with increased HbA1c (OR 1.3, 95% CI [1.2-1.4]), alongside a decline in HDL with rising HbA1c levels (P < 0.001). Elevated FBG and HbA1c are associated with higher total cholesterol and LDL levels.

**Table 2:** Lipid profile in people with different levels of FBG.

Characteristics	FBG (mg/dl)				Odds ratio (95% CI)	P value
	Q1 (21.0-114.0mg/dl)	Q2 (114.0-132.0mg/dl)	Q3 (132.0-167.0mg/dl)	Q4 (167.0-582.0mg/dl)		
Total cholesterol (mg/dl)	188.2 ( 181.9 - 194.4 )	185.7 ( 179.1 - 192.3 )	187.9 ( 181.2 - 194.7 )	192 ( 185.2 - 198.8 )	1.2 ( 1.1 - 1.3 )	<0.001
TG (mg/dl)	128.6 ( 120.3 - 136.8 )	142.7 ( 133.4 - 152.1 )	149.2 ( 139.5 - 158.9 )	167.5 ( 157.6 - 177.3 )	1.4 ( 1.3 - 1.5 )	<0.001
LDL (mg/dl)	109.6 ( 104.6 - 114.6 )	107.3 ( 101.7 - 112.8 )	107.6 ( 102.3 - 112.8 )	110.6 ( 104.7 - 116.4 )	1.1 ( 1.1 - 1.2 )	<0.001
HDL (mg/dl)	52.8 ( 51.3 - 54.4 )	49.9 ( 48.3 - 51.5 )	50.5 ( 47.2 - 53.9 )	48 ( 46.2 - 49.7 )	0.9 ( 0.8 - 1.0 )	<0.001

Using linear regression, we derived mean values and confidence intervals for fasting glucose, and lipid indices at different intervals. Multivariate logistic regression models were then used to look at the differences and risk intervals between fasting blood glucose, and lipid indices at different intervals, which included sex (male/female), ethnicity (black/Mexican/other/other Hispanic/white), blood pressure (yes/no), cigarette smoking (yes/no), alcohol (yes/no), antilipemic drugs (yes/no), antidiabetic drugs (yes/no), poverty-to-income ratio (<=1.0,1.1-3.0,>3.0), education (College/high school/Less than high school), age (continuum), BMI (continuum), FBG (continuum), Total cholesterol (continuum), TG (continuum), LDL (continuum), and HDL (continuum).

**Table 3:** Lipid profile in people with different levels of HbA1c.

Characteristics	HbA1c (%)				Odds ratio (95% CI)	P value
	Q1 (3.9-5.9.0mg/dl)	Q2 (5.9-6.5mg/dl)	Q3 (6.5-7.6mg/dl)	Q4 (7.6-17.0mg/dl)		
Total cholesterol (mg/dl)	190.5 ( 185 - 196 )	188.5 ( 182.6 - 194.4 )	182.7 ( 177.7 - 187.7 )	191.6 ( 183.9 - 199.3 )	1.2 ( 1.1 - 1.4 )	<0.001
TG (mg/dl)	131.5 ( 124.2 - 138.8 )	146.7 ( 137.8 - 155.5 )	150.8 ( 144.6 - 157 )	163.9 ( 152.1 - 175.7 )	1.3 ( 1.2 - 1.4 )	<0.001
LDL (mg/dl)	110.5 ( 106.5 - 114.5 )	109.7 ( 104.5 - 115 )	104.6 ( 99.9 - 109.3 )	109.7 ( 103.3 - 116.1 )	1.2 ( 1.1 - 1.3 )	<0.001
HDL (mg/dl)	53.7 ( 50.9 - 56.5 )	49.5 ( 48 - 51 )	48 ( 46.6 - 49.4 )	49.1 ( 47 - 51.3 )	0.9 ( 0.8 - 1 )	<0.001

Using linear regression, we derived mean values and confidence intervals for fasting glucose, and lipid indices at different intervals. Multivariate logistic regression models were then used to look at the differences and risk intervals between fasting blood glucose, and lipid indices at different intervals, which included sex (male/female), ethnicity (black/Mexican/other/other Hispanic/white), blood pressure (yes/no), cigarette smoking (yes/no), alcohol (yes/no), antilipemic drugs (yes/no), antidiabetic drugs (yes/no), poverty-to-income ratio (<=1.0,1.1-3.0,>3.0), education (College/high school/Less than high school), age (continuum), BMI (continuum), HbA1c(continuum), Total cholesterol (continuum), TG (continuum), LDL (continuum), and HDL (continuum).

### Identification of DEGs and Functional Enrichment Analysis

**Figure 4:**
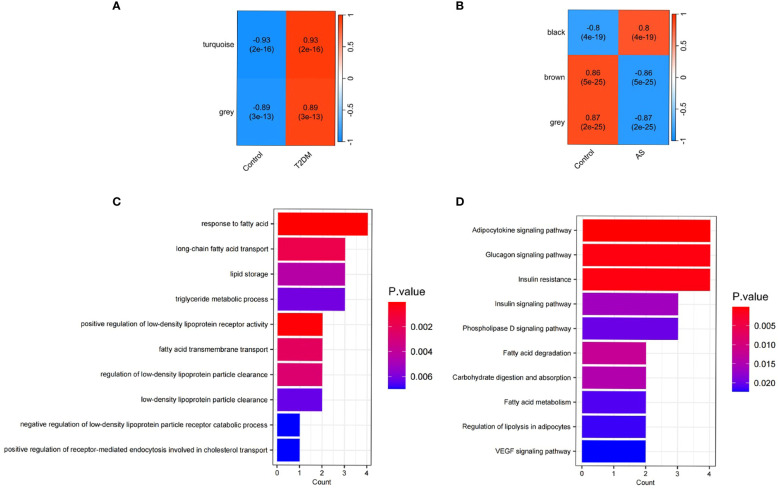
Functional enrichment analysis and WGCNA. **(A)** Correlation between modules and T2DM traits heatmap; **(B)** Correlation between modules and AS traits heatmap; **(C)** Enriched Gene Ontology (GO) terms; **(D)** Kyoto Gene and Genome Encyclopedia (KEGG) pathway.

Among the 76 common DEGs, 21 were upregulated, an increased expression level, while 21 were down-regulated, indicating a decreased expression level (**Supplementary Figures 1A-C**). GO and KEGG enrichment analyses showed that lipid metabolism-related pathways significantly enriched the differential genes in both diseases. The results of GO analysis showed that the differential genes in both diseases were increased dramatically in response to fatty acid, long-chain fatty acid transport, lipid storage, triglyceride metabolic process, positive regulation of LDL receptor activity, fatty acid transmembrane transport, regulation of LDL particle clearance, LDL particle clearance, negative regulation of LDL particle receptor catabolic process and positive regulation of receptor-mediated endocytosis involved in cholesterol transport. KEGG analysis of differential genes was mainly enriched in the Adipocytokine signaling pathway, Glucagon signaling pathway, Insulin resistance, Insulin signaling pathway, Phospholipase D signaling pathway, Fatty acid degradation, Carbohydrate digestion and absorption, Fatty acid metabolism, regulation of lipolysis in adipocytes and VEGF signaling pathway (**Figure 4D**).

### Co-expression modules of T2DM and AS analyzed by WGCNA

To construct a scale-free topological model, we chose a soft threshold β of 14 for the GSE40231 dataset and a soft threshold β of 9 for the GSE9006 dataset (**Supplementary Figures 2A-D**). These thresholds were instrumental in identifying gene modules that displayed positive associations with AS and T2DM. By applying hierarchical clustering and Spearman correlation analysis, we successfully identified three gene modules exhibiting positive associations with T2DM, encompassing 439 T2DM-related genes (**Figure 4A**). Likewise, we identified three gene modules that demonstrated positive associations with AS, encompassing 3084 AS-related genes (**Figure 4B**). Importantly, we observed 32 overlapping genes within the modules detected by the GSE40231 and GSE9006 datasets(**Supplementary Figure 1D**). These shared genes are fascinating as they may be crucial in developing AS and T2DM.

### Screening for Hub Genes

**Figure 5:**
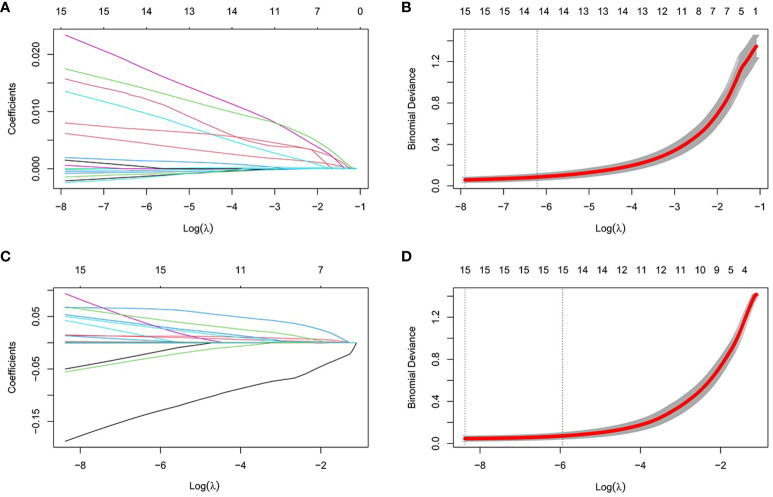
Establishment of diagnostic biomarkers by LASSO regression analysis. **(A)** LASSO coefficient profiles in T2DM; **(B)** Log (lambda) sequence used to construct a coefficient profile diagram in T2DM; **(C)** LASSO coefficient profiles in AS; **(D)** Log (lambda) sequence used to construct a coefficient profile diagram in AS.

**Figure 6:**
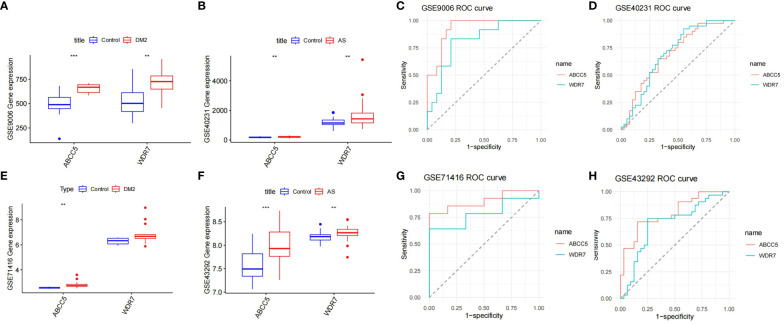
Diagnosis of genetic value. **(A)** Expression levels of the two key genes in GSE9006 in normal and T2DM patients; **(B)** Expression levels of two key genes in GSE40231 in normal and AS patients; **(C)** ROC curves of two key genes in T2DM dataset GSE9006; **(D)** ROC curves of two key genes in AS dataset GSE40231; **(E)** Expression levels of two key genes in GSE71416 in normal and T2DM patients; **(F)** Expression levels of two key genes in GSE43292 in normal subjects and AS. **(G)** ROC curves of two key genes in T2DM dataset GSE71416; **(H)** ROC curves of two key genes in AS dataset GSE43292; Box plots: X-axis represents genes, Y-axis represents expression levels. Comparison of means between groups performed by t-test (** P <0.01, *** P <0.001).

By LASSO regression analysis, 15 genes were selected as candidate genes for each of the two diseases (**Figures 5A-D**). Among them, there were six overlapping genes in both diseases. To further explore the association between these two diseases, we first tested the diagnostic effect of critical genes and whether they were differentially expressed. After removing the mismatched genes, the results showed that two genes (ABCC5 and WDR7) were significantly upregulated in T2DM and AS samples compared with standard samples (**Figures 6A, B**), and the AUC values of these two genes were more outstanding than 0.6, which provided better diagnostic effects (**Figures 6C, D**). In addition, we validated the diagnostic efficiency and expression levels of these two critical genes in validation datasets (GSE71416 and GSE43292) (**Figures 6E-H**). The results suggest that upregulation of these essential genes may lead to T2DM and induce AS.

### Changes in the proportions of immune cells in T2DM and AS

**Figure 7:**
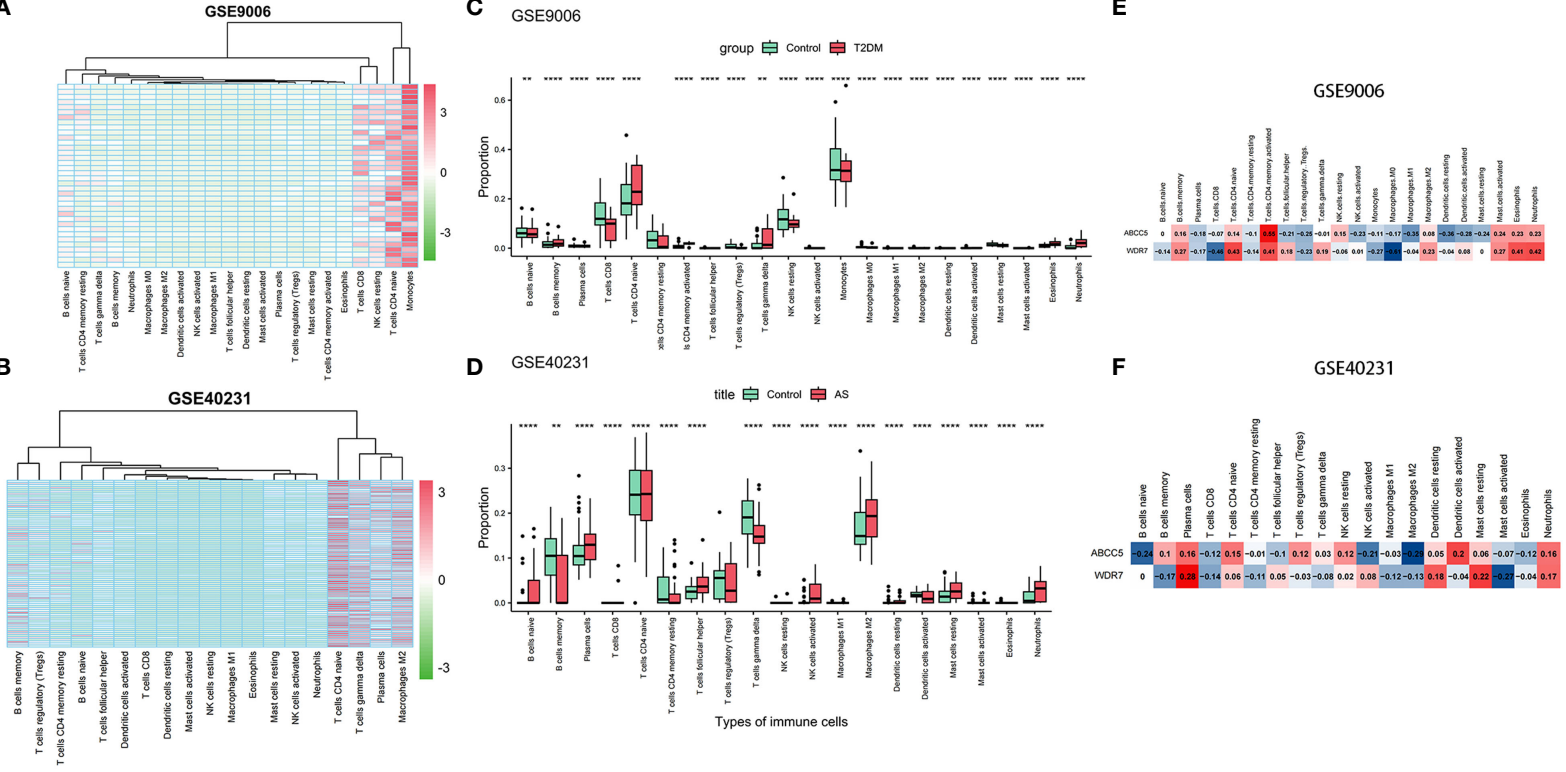
Immune infiltration analysis. **(A)** Heatmap of samples in GSE9006 dataset with immune cells; **(B)** Heatmap of samples in GSE40231 dataset with immune cells; **(C)** Infiltration in immune cells in normal and T2DM groups in GSE9006 dataset; **(D)** Infiltration in immune cells in normal and AS groups in GSE40231 dataset; **(E)** Expression of two hub genes in immune cells of GSE9006 dataset; **(F)** Expression of two hub genes in immune cells of GSE40231 dataset. (** P <0.01,**** P <0.0001).

We performed an in-depth analysis of the proportions of 22 immune cell types using the CIBERSORT algorithm. We included 12 patients with T2DM and 24 control samples, which showed a high percentage of infiltration of CD4 native T cells, resting NK cells, CD8 T cells, and monocytes. Notably, patients with T2DM demonstrated increased proportions of CD4 native T cells, gamma delta T cells, and neutrophils compared to the control group. Conversely, the proportions of CD8 T cells, resting NK cells, monocytes were decreased (**Figures 7A, C**).

Subsequently, we extended our analysis to 40 patients with AS and 40 control samples. The results demonstrated higher infiltration percentages of naive T cells,gamma delta T cells, plasma cells, and M2 macrophages among the 22 immune cell types in patients with AS. Compared to the control group, patients with AS exhibited elevated proportions of naive B cells, plasma cells, follicular helper T cells, Tregs, activated NK cells, M2 macrophages, and resting mast cells and neutrophils. In contrast, the proportions of memory B cells, resting CD4 memory T cells, and gamma delta T cells were decreased (**Figures 7B, D**). In addition, we found that two hub genes, ABCC5 and WDR7, were positively correlated with Neutrophil native cells CD4 naive, and negatively correlated with Macrophages M1 in AS and T2DM (**Figures 7E, F**).

### Identification of Therapeutic Small Molecular Agents Based on the DEGs

Based on the results obtained from the cMap database, we have identified potential small molecular agents with therapeutic implications based on the upregulated genes. Among them, the top small molecules with the highest absolute enrichment values are presented in **Table 4**, including RITA, ON-01910, doxercalciferol, topiramate. These findings provide valuable insights into the potential therapeutic options for AS and T2DM.

**Table 4:** Small molecules predicted with the common shared DEGs.

Rank	Score	Name	Description
1	98.45	RITA	MDM inhibitor
2	95.42	ON-01910	PLK inhibitor
3	92.24	doxercalciferol	Vitamin D receptor agonist
4	91.51	topiramate	Carbonic anhydrase inhibitor

## Discussion

Despite available interventions, ASCVD remains a significant cause of morbidity and mortality in individuals with T2DM (17, 18). In this study, we employed an interdisciplinary approach integrating bioinformatics, molecular biology, and clinical epidemiology to comprehensively explore the relationship between T2DM and ASCVD. In the NHANES database, we found that increases in FBG and HbA1c significantly increased the risk of elevated TG, and Ye et al. found that in patients with T2DM, elevated triglyceride levels tended to be associated with an increased risk of CVD, which may suggest that blood glucose levels play a significant role in the development of ASCVD (19) (**Tables 2**, **3**). However, the role of TG in ASCVD was not widely accepted initially, but they are now recognized as necessary (20–22).

Our identification of 76 common DEGs in both T2DM and AS patients revealed genes with abnormal expression patterns(**Supplementary Figure 1A**). Functional enrichment analysis revealed that the DEGsare significantly engaged in crucial signaling pathways governing lipid metabolism. These pathways encompass fatty acid response, long-chain fatty acid transport, lipid storage, and triglyceride metabolism, alongside the modulation of LDL receptor activity, including its positive regulation and transmembrane transport of fatty acids (**Figures 4A, B**). Moreover, our findings highlight the intricate regulation of LDL particle clearance, encompassing both its enhancement and the suppression of the receptor’s catabolic processes, as well as the facilitation of cholesterol transport via receptor-mediated endocytosis. This aligns with and substantiates the findings reported in existing literature (23, 24). This suggests that lipid metabolism plays a crucial role in the process of elevated cardiovascular disease risk in people with T2DM.

WGCNA analysis revealed ABCC5 and WDR7 as potential target genes that may play pivotal roles in the pathogenesis of both T2DM and ASCVD. Recent investigations have illuminated the pivotal role of WDR7, identified within the V-type ATPase interactome, as a crucial co-factor influencing the assembly and functional integrity of the V-type ATPase complex, essential for cellular proton (H^+^) regulation (25). Li et al (26). demonstrated that WDR7 is instrumental in modulating the assembly of the V-type ATPase. A deficiency in WDR7 triggers a compensatory expansion and subsequent over-acidification of endo-lysosomal compartments. Aberrant endo-lysosomal function could exacerbate the cellular stress response, influencing insulin signaling pathways and glucose metabolism during diabetes. Similarly, the altered intracellular trafficking and acidification may contribute to the accumulation of lipid-laden macrophages, a hallmark of atherosclerotic plaque development.

ABCC5, also known as Multidrug Resistance Protein 5 (MRP5), has been molecularly identified as the first ATP-dependent cyclic nucleotide export pump (27–29). Notably, ABCC5 mRNA is more abundant in the human heart than in other organs (30). Studies have confirmed the expression of ABCC5 at the protein level in human atrial and ventricular samples, primarily localized in vascular endothelial cells and smooth muscle cells (31). Furthermore, due to ischemic conditions, ABCC5 protein levels were upregulated in ventricular samples from patients with end-stage heart failure.ABCC5 polymorphisms have been associated with T2DM, insulin resistance, and visceral fat accumulation, indicating its potential role in damaging endothelial cells through lipid metabolic pathways (27).

Recent advancements underscore the therapeutic potential of targeted molecular interventions in addressing the complex interplay between diabetes, atherosclerosis, and their underlying mechanisms. Therefore, we screened the cMAP database for predicted small-molecule compounds (**Table 4**). RITA activates p53, thereby modulating key molecules such as HIF-1α and vascular endothelial growth factor, unveiling a new pathway that could impact metabolic diseases with pathological characteristics similar to diabetes (32). Concurrently, ON-01910inhibits Polo-like kinase 1 (Plk1), engaging in the shared molecular mechanisms of cell proliferation and inflammation, thus paving a new route for the treatment of diabetes and atherosclerosis (33). Doxercalciferol, a vitamin D receptor agonist, highlights the close association between vitamin D deficiency and conditions such as diabetes, arterial hypertension, and chronic kidney disease (34, 35). The detection of nuclear vitamin D receptors (VDRs) in vascular endothelial cells and cardiomyocytes indicates that vitamin D is directly involved in the development and progression of cardiovascular diseases (36, 37). Topiramate, promotes insulin secretion and enhances insulin sensitivity, offering an effective solution for the critical challenges of β-cell dysfunction and insulin resistance in T2DM (38).

Most of the current intractable human diseases are associated with immune system disorders, which significantly impact metabolic diseases by altering metabolism, making metabolic immunology a critical emerging discipline today. We found that the proportion of T-cell CD4 native infiltration was significantly elevated in both T2DM and AS compared to controls (P<0.001) (**Figures 7C, D**). In addition, we found that two central genes, ABCC5 and WDR7, were positively correlated with neutrophils, T cell CD4 naive, and negatively correlated with macrophage M1 (**Figures 7E, F**).

### Limitation

Firstly, the sample size and coverage of our study, although substantial, might not adequately represent the broader population affected by T2DM and ASCVD. Therefore, our sample may not capture the full spectrum of demographic and clinical variability, including age, gender, ethnicity, and comorbid conditions, which could significantly influence the disease mechanisms and outcomes. Therefore, future studies should prioritize expanding the sample size and ensuring a more diverse and representative population to enhance the external validity of the findings. the observational nature of our study inherently limits our ability to establish causal relationships between the observed variables. While we have identified correlations that suggest potential mechanisms linking T2DM and ASCVD, these associations do not imply causality. The reliance on observational data, without the ability to control for all potential confounding variables, underscores the need for cautious interpretation of the results. Experimental studies, particularly randomized controlled trials, are essential to confirm the causal links between T2DM and ASCVD and to understand the underlying biological processes.

Lastly, our research did not encompass functional experimental validation of the specific genes implicated in our findings. This limitation highlights a gap in our study, as experimental validation is crucial for verifying the biological relevance and mechanistic role of these genes in the context of T2DM and ASCVD.

Future studies should leverage more comprehensive datasets, employ methodologies that enhance data quality and representation, and incorporate experimental validations. Such endeavors will undoubtedly enrich our understanding and contribute to developing more effective strategies for the prevention, management, and treatment of T2DM and ASCVD.

## Conclusion

We identified new target genes ABCC5 and WDR7, which provide valuable avenues and directions for precision medicine and molecular mechanisms of T2DM and AS. We also proposed the potential of RITA, ON-01910, doxercalciferol, and topiramate as targeted small-molecule drugs, which marks our significant progress in precision medicine for T2DM and ASCVD.

## Data availability statement

The datasets presented in this study can be found in online repositories. The names of the repository/repositories and accession number(s) can be found in the article/**Supplementary Material**.

## Ethics statement

Ethical review and approval was not required for the study on human participants in accordance with the local legislation and institutional requirements. Written informed consent from the patients/participants or patients/participants’ legal guardian/next of kin was not required to participate in this study in accordance with the national legislation and the institutional requirements.

## Author contributions

YZ: Methodology, Formal analysis, Writing – review & editing, Visualization, Validation, Software, Data curation, Writing – original draft. LJ: Supervision, Writing – original draft, Writing – review & editing, Data curation. DY: Writing – review & editing, Methodology. JW: Writing – review & editing, Methodology, Investigation. FY: Writing – review & editing, Funding acquisition, Writing – original draft.
