# Supplementary figures and images for: Decoding cardiovascular risks: analyzing type 2 diabetes mellitus and ASCVD gene expression
Source: Front Endocrinol (Lausanne). 2024 Apr 23;15:1383772. doi: 10.3389/fendo.2024.1383772 (PMC11075663; doi:10.3389/fendo.2024.1383772)

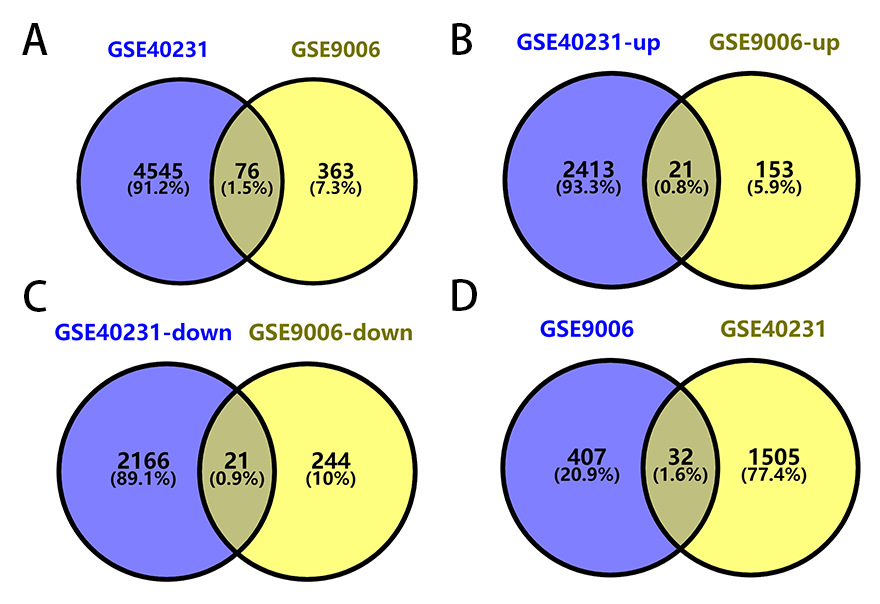

Supplement: Supplementary file 1 [file Image_1.tif]

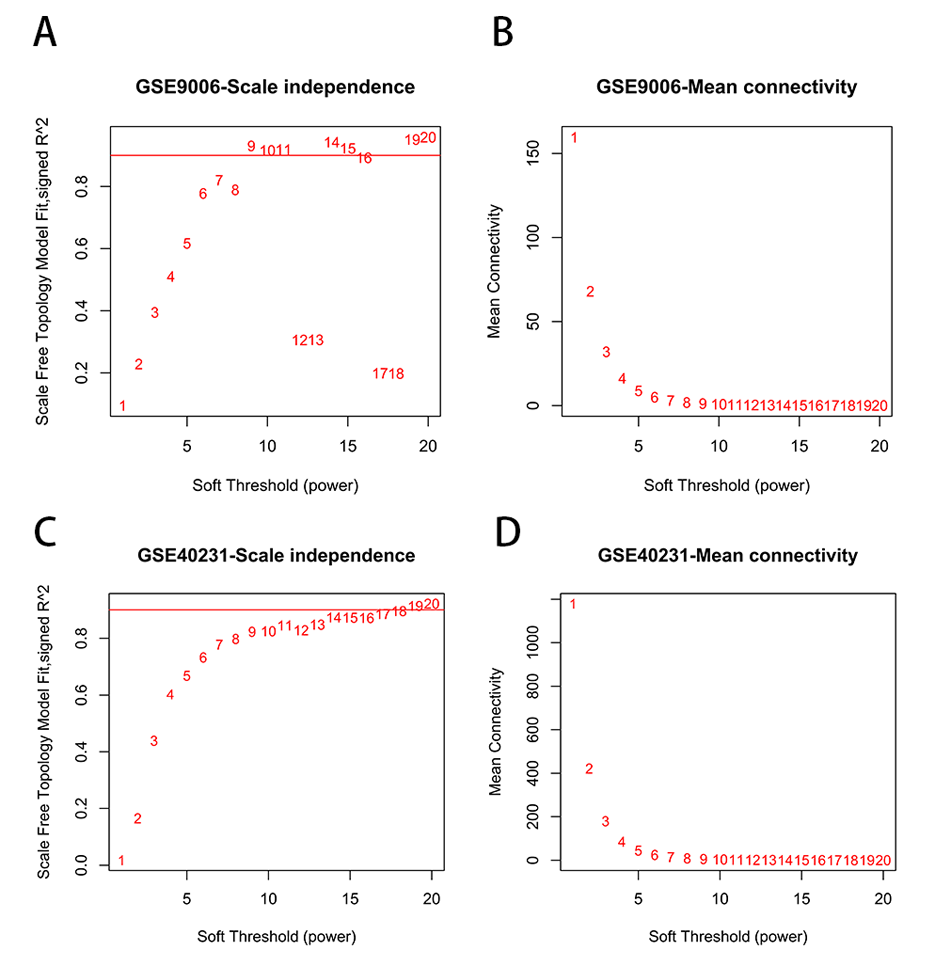

Supplement: Supplementary file 2 [file Image_2.tif]
